# Application value of antibody titres and RNA detection in the early prediction of *Mycoplasma pneumoniae* pneumonia in children: a retrospective study

**DOI:** 10.1186/s12879-023-08161-8

**Published:** 2023-04-07

**Authors:** Wenbin Tuo, Xia Guo, Mo Wu, Si Xie, Xin Shen, Jun Wang, Qinzhen Cai, Chunhui Yuan, Cong Yao, Yun Xiang

**Affiliations:** 1 Department of Laboratory Medicine, Wuhan Children’s Hospital (Wuhan Maternal and Child Healthcare Hospital), Tongji Medical College, Huazhong University of Science & Technology, Wuhan, 430016 P.R. China; 2 Health Care Department, Wuhan Children’s Hospital (Wuhan Maternal and Child Healthcare Hospital) , Tongji Medical College, Huazhong University of Science & Technology, Wuhan, P.R. China

**Keywords:** *Mycoplasma pneumoniae* pneumonia (MPP), Simultaneous amplification and testing (SAT), Particle agglutination (PA), Mycoplasma antibody (ab) titre, Sensitivity And specificity

## Abstract

**Background:**

Children with *Mycoplasma pneumoniae* pneumonia (MPP) are prone to a missed diagnosis at the early stages of the disease, which greatly affects the prognosis of children. In this study, the application value of *Mycoplasma pneumoniae* (MP) antibody titres and RNA detection for diagnosing MP infection in children with community-acquired pneumonia (CAP) was evaluated. The present study aimed to seek appropriate detection methods and strategies for early rapid diagnosis in children with MPP.

**Methods:**

A retrospective study was conducted on 563 paediatric patients aged 1 month to 15 years with CAP who were admitted to Wuhan Children’s Hospital, Tongji Medical College, Huazhong University of Science and Technology between July 2021 and February 2022. In all patients, throat swabs were collected for MP-RNA detection (simultaneous amplification and testing, SAT), and paired serum samples were collected for MP total antibody detection (particle agglutination, PA).

**Results:**

The classification as MPP or non-MPP was based on clinical diagnosis, serum MP antibody titre, and clinical or laboratory evidence of infection by other pathogen(s). Among the 563 patients with pneumonia, 187 patients were in the MPP group, and 376 patients were in the non-MPP group. The Kappa values between the particle agglutination test at different titres (1:80, 1:160) and MP-RNA detection were 0.612 and 0.660 (*P<0.01*), and the consistency of the three methods was acceptable. When the single screening method was used, MP-RNA had the highest sensitivity (93.05%), while PA (1:160) had the highest specificity (100%). PA (1:80), with an area under the curve (AUC) of 0.822, was better than PA (1:160), with an AUC of 0.783, and there was a significant difference. When the combined screening methods were used, the AUC of MP-RNA parallel PA (1:160) was significantly higher than that of titres (1:80) (z=-4.906, *P* < 0.01). Except for MP-80, the efficacy of the other three test methods in females was slightly better than that in males. Among the differences in age distribution, PA (1:80) was slightly less effective in the 13–72 months age group than at other ages, and MP-RNA parallel PA (1:160) was slightly better than the younger age group (≤ 36 m). In the older age group (> 36 m), PA (1:160) was just the opposite, while MP-RNA was slightly better than other age groups in the 13–72 months age group.

**Conclusions:**

For the diagnosis of MPP in children at the early of the disease, the antibody titre (1:160) parallel MP-RNA should be given preference, and then the disease should be further classified according to the antibody titre level and the age of the child. The combined application of the two detection methods could complement each other and strengthen the advantages, providing reliable laboratory evidence for the clinical diagnosis and timely treatment of MPP. When using the PA method alone to provide a reference standard to clarify MP infection, the differential diagnosis ability of 1:80 for MPP is better than 1:160, especially for children younger than 36 months.

**Supplementary Information:**

The online version contains supplementary material available at 10.1186/s12879-023-08161-8.

## Background

*Mycoplasma pneumoniae* (MP) is a common pathogen of acute respiratory tract infection in children throughout the world [1]. In children over five years of age, up to 40% of community-acquired pneumonia (CAP) cases are caused by MP [2]. MP infection is sporadic throughout the year, with an epidemic peak every 3–7 years [1, 3]. Compared with CAP from other aetiologies, MP-infected children are not clearly identified due to the low specificity of clinical symptoms and the lack of laboratory tests with high sensitivity and specificity [4], which results in a low initial detection rate at admission. Although *Mycoplasma pneumoniae* pneumonia (MPP) often presents as a mild and self-limiting disease, some children will develop severe pulmonary complications (e.g., obliterative bronchitis, bronchiectasis, and necrotizing pneumonia) after timely untreated, and the incidence of refractory patients is increasing yearly [1, 3]. Due to the lack of cell walls, MP is only sensitive to specific antibiotics. The disease period will be shortened, and the incidence of severe mycoplasma pneumonia will decrease if accurate antimicrobial treatment is started early in the course of the disease [4, 5]. Thus, a rapid and accurate diagnosis of MPP is critical for patient prognosis.

Expert consensus on the diagnosis and treatment of MPP in children in China points out that the diagnostic criteria include clinical manifestations and/or imaging changes of pneumonia, as well as the laboratory aetiological examination of MP, which is the most important [4, 5]. At present, laboratory detection methods for MP infection include culture, serological assays, and nucleic acid amplification tests, but all of them have several limitations [4, 5]. Culture, despite being the gold standard and carrying out strain determination, typing, and drug sensitivity tests on isolated strains, cannot be used in routine clinical practice because the cultivation environment is complicated and time-consuming [6]. Antibody (Ab) detection is the most widely used serologic test because of its fast, relatively high specificity and sensitivity in China, but it still takes a certain period before it can be detected, which may result in false negative detection [5]. In addition, antibodies can persist for a long time after the MP infection has cleared, which may result in the overuse of antibiotics [7]. Nucleic acid amplification technology, which more accurately reflects the current situation of MP infection in children, is easily affected by diverse factors, such as contamination, difficulty in obtaining high-quality samples, and the possibility of PCR inhibitors leading to false-positives or false negatives [8]. Therefore, there is an urgent need to establish a method of high sensitivity and specificity in the identification of paediatric patients with CAP who have MPP, but it is still a significant challenge.

A significant advance in laboratory diagnosis of MP infection was made when simultaneous amplification and testing (SAT) for MP-RNA detection was developed [9]. The SAT method is similar to foreign RNA diagnosis technologies, such as transcription-mediated amplification (TMA) and nucleic acid sequence-based amplification (NASBA) assays, and has been successfully applied to the detection of *Mycobacterium tuberculosis* (MTB) and enterovirus 71 (EV71) [10, 11]. SAT is based on RNA thermostatic amplification and real-time fluorescence detection technology. It takes pathogen-specific RNA (16 S rRNA) as the target and performs thermostatic amplification under the action of M-MLV reverse transcriptase and T7 RNA polymerase [10]. It has high sensitivity and specificity to allow for the rapid detection of pathogens.

However, this technology started late in the diagnosis of MP, and the relevant clinical reports are limited. Most studies mainly focus on the comparison of the overall diagnostic value [7, 12, 13], and there are few reports on the analysis of the early diagnostic value and the application prospect of combined antibody titre in screening MPP. In addition, in various expert consensuses on laboratory diagnostics of MP infection in children in China [14, 15], when the particle agglutination method is used to detect MP-Ab, a serum antibody titre ≥ 1:160 has been used as the reference standard for MP recent infection or acute infection. However, children with MPP are prone to missed diagnosis in the early stage, and some hospitals still take an antibody titre of 1:80 as the reference standard for considering MP infection in CAP children. We therefore plan to explore which antibody titre (PA 1:80 or PA 1:160) is more sensitive in the early screening of children with MPP and whether the efficiency of the combined use of the MP-RNA diagnostic method is more suitable for clinical detection.

In our study, the clinical data of CAP patients who completed paired antibody titre tests and RNA detection during hospitalization were collected. This study aimed to analyse the diagnostic performance of two detection methods at the time of admission in children hospitalized with MP pneumonia. In addition, we compared the cut-off point of antibody titres of 1:80 or 1:160 to determine which was more clinically valuable and proposed appropriate detection methods and strategies for early rapid diagnosis in children with MPP.

## Methods

### Patients

From July 2021 to February 2022, 1508 children aged 1 month to 15 years with CAP, based on the CAP management guidelines, from the Wuhan Children’s Hospital, Tongji Medical College, Huazhong University of Science and Technology were enrolled, and 945 patients without paired antibody testing and RNA detection were excluded. According to the MP diagnostic criteria [16], the remaining 563 patients were divided into the MPP and non-MPP groups. The inclusion criteria for patients with MPP were [16]: (1) Clinical diagnosis of pneumonia: fever, cough, expectoration, fast breathing (fast breathing is present when the respiratory rate is ≥ 60 breaths/min in a child aged < 2 months; ≥50 breaths/min in a child aged 2–12 months; ≥40 breaths/min in a child aged 12–60 months; ≥30 breaths/min in a child aged > 60 months), lung rales or obvious phlegm sounds, diminushed breathe sounds and chest imaging showed pulmonary infiltration and (2) Evidence of MP infection: serum MP antibody titre increased or decreased 4 times or more in convalescent or acute stage. The inclusion criteria for the patients with non-MPP were as follows [16]: (1) symptoms of respiratory infectious diseases and other types of pneumonia diagnosed clinically as non-MPP; (2) definite evidence of infection by other pathogens, such as viruses and *Streptococcus pneumoniae*; and (3) serum MP antibody titre ≤ 1:80 during hospitalization. The exclusion criteria were patients with immunosuppressive diseases, taking immunosuppressive drugs, and with underlying diseases associated with any chronic lung diseases[2]. In all the patients, throat swabs were collected for MP-RNA detection (simultaneous amplification and testing, SAT), and paired serum samples were collected for MP antibody titre detection (particle agglutination, PA).

## Data collection

This retrospective study collected data from 563 patients at Wuhan Children’s Hospital. To avoid missing any patients, MP test medical order records were also reviewed. Demographic, laboratory, serological test data and MP-RNA detection results were extracted using a standardized data-collection sheet and were secured by authorization and encryption. The chief complaints and demographic characteristics were extracted from electronic medical records (EMRs); laboratory data (such as PCT, WBC, PLT, etc.), serological test data and MP-RNA detection results were acquired from the LIS (Laboratory Information System). Those data were recorded and quality controlled by the clinical data repository, and CRFs (Patient Report Form) were provided. Parallel logical examinations on the data extracted from the CRF were performed by two skilled doctors. All patients, both included and excluded, were reviewed by the research team.

## Ethics statement

This research was approved by the institutional ethics board of Wuhan Children’s Hospital (Wuhan Maternal and Child Healthcare Hospital), Tongji Medical College, Huazhong University of Science & Technology (NO.: 2022R048-E01). All methods and experimental protocols in this study were conducted in accordance with the approved protocols and Ethics Committee’s existing guidelines.

## Throat swab

A sterile cotton swab was rubbed with rotation over 1 tonsillar area, then the arch of soft palate and uvula, the other tonsillar area, and finally the posterior pharyngeal wall. The head of the throat swab was broken into a vial containing 1 mL of phosphate buffered saline (PBS) buffer used as transport medium and transported to the laboratory after proper labelling of the vial. In the laboratory, the swabs were twirled thoroughly, expressed against the sides of the tube, and discarded. The samples were stored at − 70 °C until PCR analysis [5] was performed.

## Serological test

The acute phase sera were collected from the patients in this study during the first 24 h of admission. Convalescent phase sera were obtained after 1–3 weeks of enrolment. All serum samples were preserved at − 20 °C until detection of antibodies against MP. The detection of IgM and IgG antibodies against MP in the serum was determined using the passive particle agglutination assay (SERODIA-MYCO II, No.: YZB/JAP8033-2013, Japan), according to the recommendations of the manufacturer. The results are reported as antibody titres, with titres of < 1:40, 1:80, 1:160, and 1:320.

## SAT for MP-RNA detection

Throat swab specimens of the children were collected and tested using an MP nucleic acid assay kit (ZhongZhi Biotechnologies, Wuhan, China), and the tests included nucleic acid extraction and isothermal amplification detection. The collected pharyngeal swabs were first mixed, centrifuged, pretreated, dissolved in normal saline, added to the same amount of cell lysate, and lysed at room temperature for 5–10 min to prepare the samples to be tested. Two microlitres of the sample to be tested and 17 µL of the amplification reaction solution were added to the PCR tube. The samples were incubated at 95 ℃ for 2 min in the PCR instrument. After the temperature dropped to 42 ℃ and was stabilized for 2 min, 1 µL amplification enzyme was added and incubated at 42 ℃ for 90 min. The final amplified RNA products were qualitatively analysed by biotin signal amplification technology.

One positive quality control (QC) test was used as the positive control, and two negative QC tests were used as the negative controls. The result was determined as follows: the relative light unit (RLU) of the three test indicators of the sample and the negative and positive QC were read, and each test indicator corresponded to one RLU of positive QC and two RLUs of negative QC. When the average RLU of the two negative QC materials corresponding to the test indicator was < 1000, the ratio R of the sample to be tested = the RLU of the sample to be tested/ (10 ⋅ the average RLU of the negative QC material). When the average RLU of the two negative QC materials corresponding to the test indicator was ≥ 1000, the ratio R of the sample to be tested = the RLU of the sample to be tested/ (5 ⋅ the average RLU of the negative QC material). Qualitative judgement was made on the sample according to the ratio R, and if the ratio R >1.0, it was judged as positive, and if it was ≤ 1.0, it was negative.

### Statistical analysis

R package (4.0.2) was used for all statistical analyses. According to the data type, descriptive analysis such as constituent ratio was carried out for the frequency data such as gender and MP-RNA, and median IQR was used for the continuous data such as hsCRP according to its distribution. The Mann-Whitney U test was used to test the distribution of hsCRP and other blood test parameters between the different groups, and the matched McNemar test was used to test the effectiveness of the different diagnostic methods. The receiver operating characteristic (ROC) curve was used to explore the value of each variable in the diagnostic model, Clarke Pearson and Delong methods were used to test the difference in the area under the ROC curve (AUC) between the different diagnostic methods, the Breslow-Day test and logistic regression model were applied to analyse the influence of confounding factors on the diagnostic efficiency, and a decision tree model was used to screen the optimal diagnostic process. Sankey diagrams were applied to display the effects of applying the different detection methods at different stages. P < 0.05 was statistically significant.

## Results

### Clinical characteristics of patients

A total of 1508 CAP patients were enrolled in this study. Because the analysis focused on the diagnostic efficacy of MP-RNA detection and antibody agglutination testing, the 945 patients without uncompleted paired antibody testing and RNA detection were excluded. Of the remaining 563 patients (Table 1), 187 (33.2%) were considered MPP patients; 376 (66.8%) were non-MPP patients who were finally diagnosed with bacterial or viral pneumonia. The characteristics of the participants are shown in Table 1. Based on the basic characteristics, there was no difference in the distribution proportion between males and females, but there were age differences. The age of the MPP group was significantly older than that of the non-MPP group, and the difference was statistically significant. Based on the laboratory indicators, there was no significant difference in PCT, WBC, PLT, EOS, and BASO between the two groups, but the other indicators were different. The patients in the MPP group had some indicators, such as hsCRP, RBC, HGB, HCT, and NEU, which were significantly higher than those of the patients in the non-MPP group, and others, such as LYM and MONO, were the opposite. The *P value*s were all < 0.01.

**Table 1:** The clinical data of the study population (n/IQR, P)

Characteristic	Total (n = 563)	Non-MPP (n = 376)	MPP (n = 187)	*P*
Gender Male	332	225	107	0.552
Female	231	151	80	
Age (M)	36 [12, 60]	24 [12, 48]	48 [24, 84]	< 0.01
hsCRP (mg/L)	5.1 [0.78, 22.1]	4.05 [0.78, 20.2]	8.19 [2.42, 27.83]	< 0.01
PCT (ng/mL)	0.1 [0.06, 0.23]	0.1 [0.06, 0.23]	0.09 [0.06, 0.19]	0.185
WBC (10^9^/L)	8.15 [5.98, 10.76]	8.22 [5.92, 10.83]	7.6 [6.31, 9.91]	0.447
RBC (10^12^/L)	4.49 [4.21, 4.72]	4.49 [4.23, 4.7]	4.56 [4.28, 4.85]	< 0.01
HGB (g/L)	121 [114, 128]	121 [113, 127]	124 [117, 131]	< 0.01
HCT (%)	36.7 [34.6, 38.7]	36.7 [34.7, 38.6]	37.6 [35.3, 39.7]	< 0.01
PLT (10^9^/L)	310.5 [250.25, 400]	308 [251, 396]	296 [237, 398]	0.317
NEU (10^9^/L)	3.42 [2.08, 5.52]	3.14 [1.86, 5.29]	4.16 [3.04, 5.77]	< 0.01
LYM (10^9^/L)	3.11 [2.02, 4.71]	3.36 [2.21, 5.02]	2.45 [1.78, 3.43]	< 0.01
MONO (10^9^/L)	0.59 [0.38, 0.85]	0.61 [0.38, 0.87]	0.52 [0.39, 0.73]	< 0.01
EOS (10^9^/L)	0.1 [0.02, 0.23]	0.09 [0.02, 0.24]	0.1 [0.03, 0.23]	0.344
BASO (10^9^/L)	0.02 [0.01, 0.03]	0.02 [0.01, 0.03]	0.01 [0.01, 0.02]	0.054

MPP: *Mycoplasma pneumoniae* pneumonia; hsCRP: hypersensitive-c-reactive-protein; PCT: procalcitonin; WBC: white blood cell; RBC: red blood cell; HGB: haemoglobin; HCT: haematocrit; PLT: platelet; NEU: Neutrophil Count; LYM: lymphocyte; MONO: monocyte; EOS: eosinophil; BASO: basophil

At the same time, due to the different time intervals of MP-RNA and MP-Ab detection from onset to hospitalization in different children, we divided the detection time intervals into four groups: ≤ 7 days, 8 ~ 14 days, 15 ~ 30 days, and > 30 days, according to the characteristics that IgM antibody generally appeared 4–5 days after infection and reached the peak time point in 3–4 weeks. The detection time distribution of the study population is shown in Table S1. The distribution of the MP group (> 30 d) was significantly lower than that of the non-MP group, and the *P value*s were all 0.005, which means that the difference was statistically significant.

## Correlation between MP-RNA and particle agglutination assay (1:80/1:160) detection results at admission

The diagnostic efficacy of the particle agglutination assay at different titres (1:80, 1:160) and MP-RNA was tested by the McNemar test, and the differences were statistically significant, with all the *P value*s < 0.01 (Table 2). The Kappa values of the particle agglutination test and MP-RNA detection at different titres (1:80, 1:160) were 0.612 and 0.660, respectively, indicating that the consistency of the three methods was acceptable.

**Table 2:** Comparison of diagnostic efficacy between the MP-Ab (PA) and MP-RNA (n, P) at admission

MP-Ab (PA)		MP-RNA		Kappa	p
		Negative	Positive		
(1:80)	Negative	340	50	0.612	< 0.01
	Positive	44	129		
(1:160)	Negative	377	80	0.600	< 0.01
	Positive	7	99		

PA: particle agglutination

## Diagnostic value of different methods using paired ab titres as a diagnostic standard

We compared the diagnostic values of MP-RNA, PA (1:80), and PA (1:160), considering paired Ab titres as the standard. Table 3 shows that when the single screening method was used, MP-RNA had the highest sensitivity (93.05%), while PA (1:160) had the highest specificity (100%). The difference in the AUC of the three methods is statistically significant by the Clarke Pearson test, *P* < 0.01. Delong analysis was used between the three methods, and the results of pairwise comparison with the DeLong method showed that the Z values of MP-RNA, PA (1:80), and PA (1:160) were 6.77, 8.54, 2.48, all *P* < 0.01, respectively. The differences were statistically significant. The above results shows that MP-RNA is a better method under separate test.

**Table 3:** Comparison of sensitivity, specificity, accuracy, kappa, Youden and AUC values of Mycoplasma pneumoniae different detection methods using paired Ab titres as a diagnostic standard

Methods	Sensitivity	Specificity	Accuracy	Kappa	Yourden	Auc
MP-RNA	93.05%	98.67%	96.80%	0.927	0.917	0.959
PA (1:80)	73.80%	90.69%	85.08%	0.657	0.645	0.822
PA (1:160)	56.68%	100.00%	85.61%	0.636	0.567	0.783
MP-RNA parallel PA (1:80)	97.86%	89.36%	92.18%	0.832	0.872	0.937
MP-RNA parallel PA (1:160)	96.79%	98.67%	98.05%	0.956	0.955	0.977

PA: particle agglutination

When the combined screening methods were used, the diagnostic efficacy of MP-RNA parallel PA (1:80, 1:160) was analysed by Delong methods, and the results showed that the AUC of MP-RNA parallel PA (1:160) was significantly higher than that under titre (1:80), z=-4.906, *P* < 0.01. MP-RNA and MP-RNA parallel PA (1:160) were used as the better methods in the separate test and the combined test, respectively. The AUCs of the two methods were compared by the Delong method. The results showed that MP-RNA parallels PA (1:160) was better than MP-RNA, z = 2.690, *P* = 0.007. PA (1:160) has good differential diagnostic value because its specificity is 100%.

## Comparison of the effects of different confounding factors on the efficacy of four diagnostic methods

To avoid the influence of confounding factors in Table 1 and Table S1 on the four methods, ROC model Formula Y = D X (Y = c (MP-RNA, MP-80, MP-160, MP-RNA parallel PA (160)), D = MP, X = covariate), using logit as the linking function, the efficacy of the four test methods were evaluated. The Breslow‒Day test was used to test the effect of gender on the four test methods. The results showed that there was no significant difference in the four methods between males and females (*P* > 0.05). Because the specificity of the PA (1:160) method was 100%, there was no difference between males and females. The rest of the results showed that except for the effect of age on the efficacy of the MP-RNA test (*P* < 0.01), the other confounding factors did not affect the four test methods (Table 4). Thus, there is only a difference in the efficacy of the MP-RNA test among different age groups.

**Table 4:** Influence of different confounding factors on the efficacy of four diagnostic methods

Covariate	MP-RNA			PA (1:80)			PA (1:160)			MP-RNA parallel PA (1:160)	
	${\chi }^{2}$/*β*	*p*		${\chi }^{2}$/*β*	*p*	${\chi }^{2}$/*β*		*p*		${\chi }^{2}$/*β*	*p*
gender	0.746	0.388		1.802	0.179	-		-		0.11	0.74
Age group	0.447	0		0.094	0.699		0.225	0.132		0.063	0.843
RNA detection time	-0.439	0.098		0.19	0.133		0.212	0.217		-0.420	0.216
MP-Ab detection time	-0.460	0.076		0.244	0.05		0.256	0.133		-0.419	0.210
hsCRP (mg/L)	-0.004	0.342		0.02	0.091		-0.009	0.182		-0.009	0.182
RBC (10^12^/L)	0.077	0.768		-0.328	0.524		-0.569	0.079		-0.390	0.545
HGB (g/L)	0.013	0.214		-0.008	0.703		-0.013	0.312		-0.019	0.447
HCT (%)	0.035	0.318		-0.044	0.528		-0.069	0.105		-0.061	0.481
NEU (10^9^/L)	0.033	0.974		-0.001	0.974		-0.002	0.964		0.137	0.073
LYM (10^9^/L)	-0.023	0.689		-0.206	0.072		0.097	0.233		-0.081	0.562
MONO (10^9^/L)	-0.682	0.067		0.749	0.234		-0.117	0.822		0.926	0.154

PA: particle agglutination; hsCRP: hypersensitive-c-reactive-protein; RBC: red blood cell; HGB: haemoglobin; HCT: haematocrit; PLT: platelet; NEU: Neutrophil Count; LYM: lymphocyte; MONO: monocyte

## Comparison of the efficacy of four diagnostic methods under different sexes and ages

Furthermore, we explored the effectiveness of the four test methods under different ages and sexes (Table 5). The results showed that except for MP-80, the efficacy of the other three test methods in females was slightly better than that in males. In terms of age distribution, PA (1:80) was slightly less effective in the 13–72 months age group than at other ages, MP-RNA parallel PA (1:160) was slightly better than the younger age group (≤ 36 m) in the older age group (> 36 m). PA (1:160) was just the opposite, while MP-RNA was slightly better than other age groups in the 13–72 months age group. This shows that age has certain guiding significance for children to choose which detection method.

**Table 5:** Comparison of the efficacy of four diagnostic methods for different sexes and ages

Variable	Category	Auc (SE (A))						
		MP-RNA		PA (1:80)		PA (1:160)		MP-RNA parallel PA (1:160)
Gender	Male	0.947 (0.017)		0.829 (0.028)		0.776 (0.032)		0.975 (0.012)
	Female	0.975 (0.013)		0.812 (0.032)		0.794 (0.036)		0.981 (0.011)
Age group (M)	1 ~ 12	0.953 (0.035)		0.829 (0.059)		0.783 (0.066)		0.996 (0.004)
	13 ~ 36	0.974 (0.016)		0.791 (0.041)		0.722 (0.047)		0.993 (0.006)
	37 ~ 72	0.957 (0.022)		0.787 (0.043)		0.810 (0.042)		0.966 (0.020)
	> 72	0.934 (0.031)		0.816 (0.049)		0.817 (0.045)		0.953 (0.027)

M: months

## Classification effect of step-by-step diagnosis with different screening methods in children with MP

**Fig. 1:**
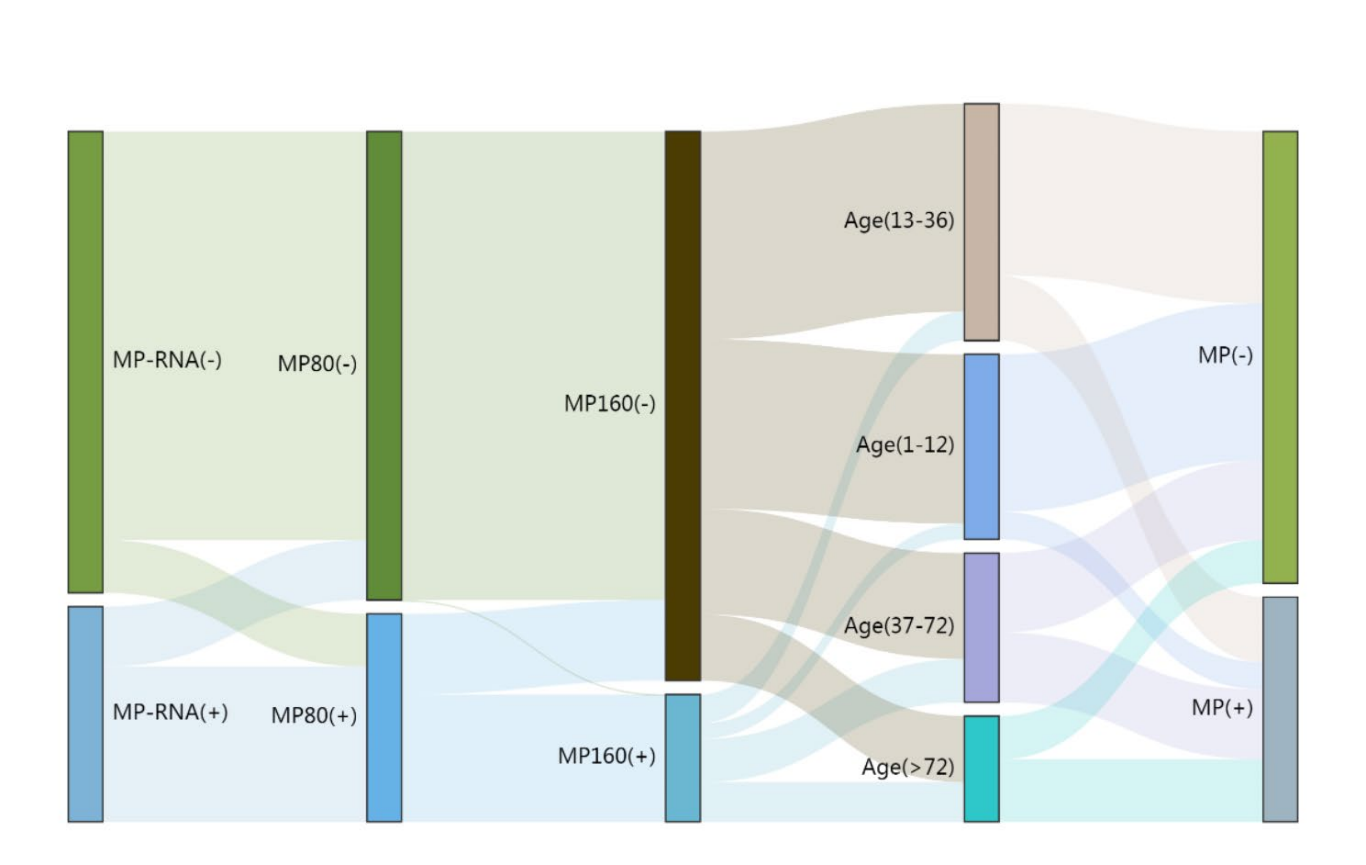
Sankey diagram for the diagnosis of MP in children. MP-RNA, MP-80, and age had a good discriminative effect on the shunt of children with MP

According to the frequency distribution table of children with MP-RNA, PA (1:80), PA (1:160), age, and final diagnosis of MP (Table S2), we drew a Sankey diagram online using a highchart. The triage effect of the step-by-step diagnosis of different screening methods in children with MP is displayed in Fig. 1. Among 187 children with MP, the MP-RNA parallel PA (1:160) was used preferentially. A total of 181 patients were found, with a positive predictive value of 97.31% and a negative predictive value of 98.41%. The overall accuracy was 98.05%. After that, it could be classified twice according to age, MP-RNA, and antibody titre. For patients with negative MP-RNA parallel PA (1:160), if the age was ≤ 36 m, they were all true negatives. The false-negative rate was 5.66% when the age was more than 36 m. For the patients with positive MP-RNA parallel PA (1:160), if the antibody titre was ≥ 1:80, they were all true positives, while the false-positive rate of antibody titre < 1:80 was 10% (Figure S1). Overall, MP-RNA, MP-80, and age had a good discriminative effect on shunts in children with MP.

**Fig. 2:**
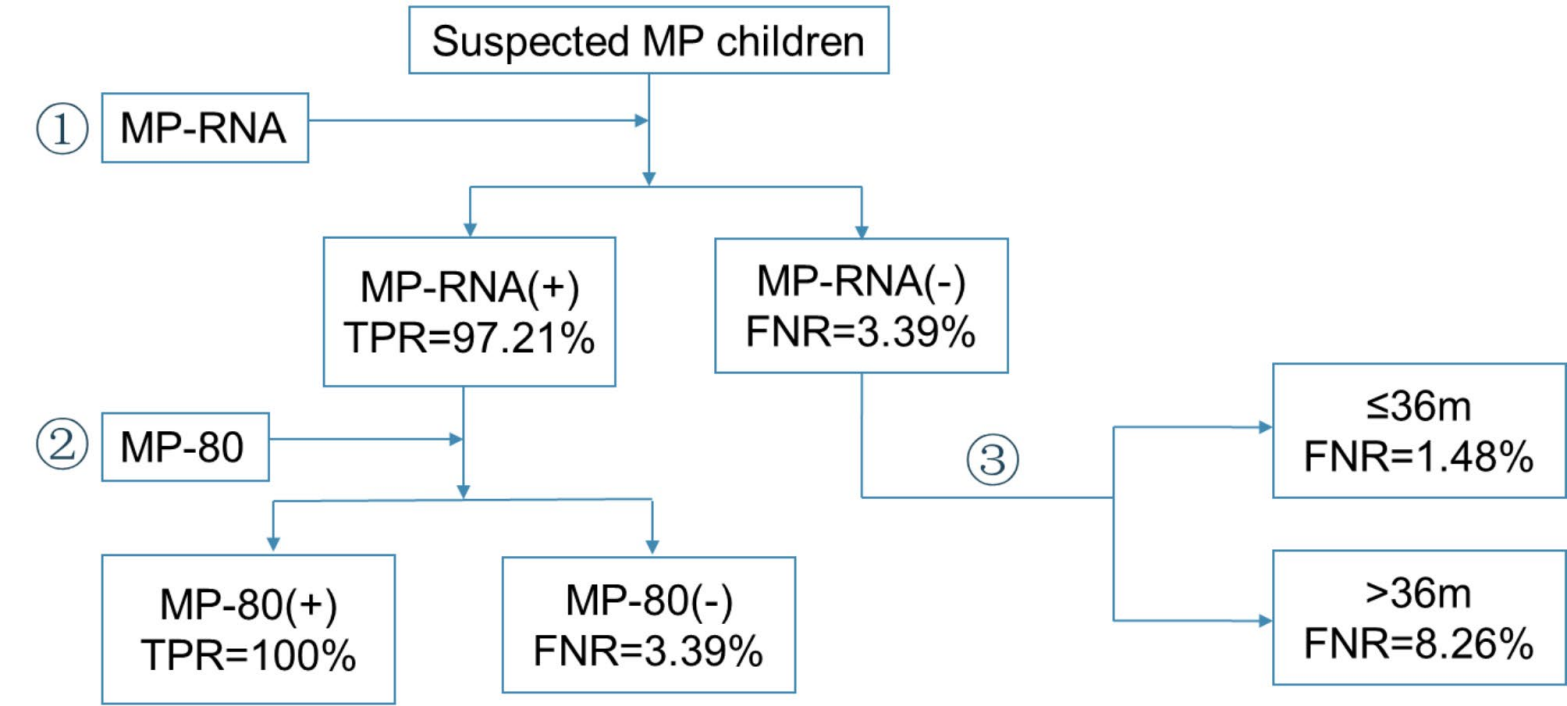
Diagnostic flow chart of children with MP. TPR = true positive rate, FNR = false negative rate

Combined with the Sankey diagram (Fig. 1**)** and decision tree model (Figure S1), we have made the diagnosis flow chart (Fig. 2**)** of MP children for the convenience of application. All suspected MP children were tested for MP-RNA. According to the test results, the true positive rate of MP-RNA (+) children was 97.21%. To further determine that MP-80 can be tested, the diagnostic rate of all MP-80 (+) children was 100%, while the false negative rate of MP-80 (-) children was 3.39%. The false negative rate of MP-RNA (-) children was 3.39%. To further determine the difference according to the age of children, the false negative rate of all (≤ 36 m) children was 1.48%, while the false negative rate of (> 36 m) children was 8.26%.

## Discussion

MPP and CAP caused by other aetiologies share highly similar clinical, radiological, and histopathological characteristics, leading to great challenges in the differential diagnosis in the early stages of illness [3, 4]. The therapies for MPP and non-MPP differ greatly [1, 17]. How to choose early, fast and accurate diagnostic methods is a difficult problem for laboratory diagnosis.

In this study, we collected the results of MP-RNA and MP-Ab in 563 patients with CAP, and systematically evaluated the three methods. Our investigation showed that the consistency of single MP-Ab under different titres (1:80, 1:160) and MP-RNA on the first day of hospitalization were acceptable (Table 2). Similar with the Ren YanHong et al. reported [18]. However, 16.7% and 15.4% of the children had inconsistent diagnostic results in the comparison of different antibody titres (1:80, 1:160) and MP-RNA, respectively. The reasons for the inconsistent results may include the following: first, there is a window in the detection of serum MP-Ab because the antibody generally appears one week after infection and reaches a peak at 3–4 weeks [14]. In the early stage of the disease, the antibody titre does not rise, and the tests are prone to false negative. Second, MP-SAT takes nucleic acid RNA as the target and detects MP through efficient amplification technology, which can make up for the window problem of serological detection. However, high-quality samples for RNA detection are difficult to obtain.

At the same time, we found that the diagnostic effectiveness of the two methods were inconsistent (Table 3). When the single screening method was used, our results shows that SAT had good diagnostic accuracy in children with MPP compared with the PA, with a sensitivity of 93.05%. However, some children will still be misdiagnosed. One explanation for the “false-negative” SAT results might be that the children were treated with long-term antimicrobial treatment, which may decrease the MP RNA load [7]. Another potential explanation is related to the pathogenesis profile of MP. The acute lung injury that occurs may not necessarily be associated with MP colonization of the lung in MPP patients. Instead, hyperimmune reactions by the host’s immune system in response to the infection may also lead to PCR negativity [9]. This shows that the application of the two methods in the early diagnosis MP had different limitations.

In addition, the expert consensus on the diagnosis of MP infection in China recommends that the titre of MP-specific antibody ≥ 1:160 in a single serum test is the basis for the diagnosis of MP infection [15]. Although the specificity of PA (1:160) was 100%, the ROC curve in this study shows that this threshold was slightly higher than the best diagnostic point (Table 3), which easily causes a missed diagnosis. Our results showed that PA (1:80) had better diagnostic ability than PA (1:160) for the differentiation of MPP. In actuality, one study with 433 paediatric patients found that the average number of disease days with an antibody titre of PA (1:160) was 5.89 d, so for patients within 1 week of onset, using the single antibody titre (1:60) of the PA antibody titre as the diagnostic standard was easy to cause false negative results [18]. Therefore, we do not recommend PA (1:160) as the reference standard.

Our current research results and conclusions reported by other studies both show that the effect of a single diagnostic method may be limited [5, 7, 19]. Although molecular assays are superior in detecting MP during the early periods, they cannot take the place of serology. A serum MP antibody titre that is increased or decreased 4 times or more in the convalescent or acute stage is still used as a final diagnosis for children with MPP [13]. To explore the value of the combination of the two diagnostic methods in differentiating MPP infection in children, the combined screening effect of MP-RNA and PA at different titres was evaluated, and the diagnostic efficiency of the MP-RNA parallel antibody titre (1:160) was better than that of MP-RNA alone. This showed that MP-SAT plus serology could be a good screening test for the reliable and accurate diagnosis of MP. In addition, when MP-RNA and MP-Ab were used together, the results were analysed as follows: 1). Double-positive results: MP infection can be basically diagnosed. In such patients, the onset time is more than one week, and the antibody has been produced. RNA detection can be used in the later observation of curative effects [14]. 2). Single positive result: the serological test result is negative, and the molecular biology test result is positive: an early MP infection can basically be diagnosed. First, the onset time is short, and the antibody has not been produced. Second, the development of the immune system is imperfect, immune function is low, no antibody is produced or the antibody titre is low. The 4-fold change in antibody titre levels can further confirm MP infection [14]. The serological test results are positive and molecular biology test results are negative: the current or previous infection of MP needs to be judged according to the positive type and titre of the antibody, and these cases are mostly due to previous infection after treatment [14]. 3). Double negative results: the possibility of MP infection is very small. When a further differential diagnosis is needed, the antibody titre can be monitored or resampled [14].

We previously analysed the basic characteristics and laboratory indicators of the included children, as well as the detection time distribution of the two test methods. After eliminating the influence of confounding factors on the effectiveness of the four test methods, we found that there were differences in the efficacy of MP-RNA testing among different age groups (Table 4). The study population was further grouped by age, and the results showed that the diagnostic efficiency of the combined use of MP parallel PA (1:160) was better than that of the other three methods at any age, which further confirmed that the application value of the combined use was better than that of the single use. When a method was used alone, the diagnostic efficiency of MP-RNA was better than that of PA. This suggested that when a method was selected, MP-RNA should be chosen first, which was consistent with previous conclusions [6, 8, 12, 20]. But we still found that the age of the confounding factors had the least influence on the combined method.

We also compared and analysed the diagnostic performance of 1:80 and 1:160 separately. The results showed that although the specificity of 1:160 was 100%, in general comparison, the AUC of 1:80 was better than that of 1:160. To determine the reason, we further subdivided the age of children with the only influencing factor. The result analysis showed that in the young age group (< 36 m), the AUC value of 1:80 was better than that of 1:160, and the younger the age, the more obvious it was. In the older age group, there was almost no difference in the AUC value among the children over 6 years old. The possible reasons were that the immune function of the body is not fully established in young children, and sufficient antibodies cannot be produced in time against MP infection [1]. However, children > 36 m have a certain resistance, and it is speculated that carrying the MP organism may be involved [3]. Thirty-six months old is a better time node to judge the gradual maturity of the cellular immune function of infants. Children under the age of 36 m have difficulty discharging secretions due to the narrowing of the respiratory cavity and the narrowing of the pressure [20]. These children have increases in secretions within the pharynx, and the secretions are more viscous. When sampling with a pharyngeal swab, it is easy to cause false negative results due to uneven sampling.

In summary, if a single screening method is used, MP-RNA should be given priority. There are two reasons: (1) Under a single screening method, the specificity and sensitivity are relatively high. (2) Compared with MP-RNA + MP Ab testing, MP-RNA costs less. If combined screening is used, the MP-RNA parallel antibody titre (1:160) should be given preference, and then the disease should be further classified according to the antibody titre (1:80) level and the age of the child (36 m). In the absence of RNA detection conditions, PA is commonly used to detect antibody titre levels, and the differential diagnosis ability of 1:80 for MPP is better than that of 1:160, especially for children younger than 36 m. The above seems to be the appropriate strategy for the early diagnosis of MP infection.

The present study has some limitations. One is that the sample size was relatively small and all participants were from a single centre. it is recommended to further expand the sample size for evaluation. And another is that we compared the two methods of RNA detection and total antibody (IgG and IgM), and lack of comparison with the commonly used DNA detection methods and single IgM methods in MP.

## Conclusions

In view of the unique aetiological characteristics of MP, the problem of qualified materials for aetiological detection, the time window of serological detection and the influence of immune conditions, as well as the different sensitivity and specificity of different detection methods, the final diagnosis cannot be achieved by using one detection method. However, there is a good prognosis following early diagnosis and treatment. Therefore, the joint diagnosis of molecular biology and serology is still the diagnostic strategy for MP infection advocated at present. The MP-RNA parallel antibody titre (1:160) should be given preference in patients, for different results, diseases can be further classified according to antibody titer (1:80) level and child age (36 m). The combined application of the two detection methods could complement each other and strengthen the advantages, providing reliable laboratory evidence for the clinical diagnosis and timely treatment of MPP. In addition, when using the PA method alone to provide a reference standard to clarify MP infection, the differential diagnosis ability of 1:80 for MPP is better than 1:160, especially for children younger than 36 m. However, we felt a verification experiment with a larger sample size should be used in future work.

## Electronic supplementary material

Below is the link to the electronic supplementary material.

Table S1. The detection time distribution of the study population (n, P).

Table S2. Frequency table of diagnosis distribution in children under different diagnostic methods and age groups.

Figure S1. Diagnostic decision trees for children with MP.

## Data Availability

The datasets used and/or analysed in the study are available from the corresponding author on reasonable request.

## Abbreviations

SAT: simultaneous amplification and testing
PA: particle agglutination
MPP: *Mycoplasma pneumoniae* pneumonia
AUC: area under the curve
PPV: positive predictive value
MP, *M. pneumoniae*: *Mycoplasma pneumoniae*
CAP: community-acquired pneumonia
Ab: antibody
TMA: transcription-mediated amplification
NASBA: nucleic acid sequence-based amplification
EV71: enterovirus 71
hsCRP: hypersensitive-c-reactive-protein
PCT: procalcitonin
WBC: white blood cell
RBC: red blood cell
HGB: haemoglobin
HCT: haematocrit
PLT: platelet
NEU: neutrophil count
LYM: lymphocyte
MONO: monocyte
EOS: eosinophil
BASO: basophil
