# Supplementary material for: Application value of antibody titres and RNA detection in the early prediction of Mycoplasma pneumoniae pneumonia in children: a retrospective study
Source: BMC Infect Dis. 2023 Apr 7;23:220. doi: 10.1186/s12879-023-08161-8 (PMC10082536; doi:10.1186/s12879-023-08161-8)
Supplement: Supplementary file 1 — Table S1. The detection time distribution of the study population (n, P). [file 12879_2023_8161_MOESM1_ESM.docx]

**Table S1.** The detection time distribution of the study population (n, *P*).

| Characteristic | Total (n=563) | Non-MPP (n=376) | MPP (n=187) | *P* |
| --- | --- | --- | --- | --- |
| RNA detection time |  |  |  | 0.005 |
| ≤7d | 241 (42.81) | 175 (46.54) | 66 (35.29) |  |
| 8～14d | 193 (34.28) | 112 (29.79) | 81 (43.32) |  |
| 15～30d | 74 (13.14) | 47 (12.5) | 27 (14.44) |  |
| >30d | 55 (9.77) | 42 (11.17) | 13 (6.95) |  |
|  |  |  |  |  |
| MP-Ab detection time |  |  |  | 0.005 |
| ≤7d | 269 (47.78) | 193 (51.33) | 76 (40.64) |  |
| 8～14d | 169 (30.02) | 96 (25.53) | 73 (39.04) |  |
| 15～30d | 71 (12.61) | 46 (12.23) | 25 (13.37) |  |
| >30d | 54 (9.59) | 41 (10.9) | 13 (6.95) |  |
