# Supplementary material for: Application value of antibody titres and RNA detection in the early prediction of Mycoplasma pneumoniae pneumonia in children: a retrospective study
Source: BMC Infect Dis. 2023 Apr 7;23:220. doi: 10.1186/s12879-023-08161-8 (PMC10082536; doi:10.1186/s12879-023-08161-8)
Supplement: Supplementary file 2 — Table S2. Frequency table of diagnosis distribution in children under different diagnostic methods and age groups. [file 12879_2023_8161_MOESM2_ESM.docx]

**Table S2.** Frequency table of diagnosis distribution in children under different diagnostic methods and age groups.

| Type | MP-RNA | PA (1:80) | PA (1:160) | Age group | MP | F |
| --- | --- | --- | --- | --- | --- | --- |
| 1 | MP-RNA (-) | MP80 (-) | MP160 (-) | (37~72) | MP (+) | 2 |
| 2 | MP-RNA (-) | MP80 (-) | MP160 (-) | >72 | MP (+) | 2 |
| 3 | MP-RNA (-) | MP80 (+) | MP160 (-) | (37~72) | MP (+) | 2 |
| 4 | MP-RNA (-) | MP80 (+) | MP160 (+) | (1~12) | MP (+) | 2 |
| 5 | MP-RNA (-) | MP80 (+) | MP160 (+) | (13~36) | MP (+) | 2 |
| 6 | MP-RNA (-) | MP80 (+) | MP160 (+) | (37~72) | MP (+) | 1 |
| 7 | MP-RNA (-) | MP80 (+) | MP160 (+) | >72 | MP (+) | 2 |
| 8 | MP-RNA (+) | MP80 (-) | MP160 (-) | (1~12) | MP (+) | 7 |
| 9 | MP-RNA (+) | MP80 (-) | MP160 (-) | (13~36) | MP (+) | 18 |
| 10 | MP-RNA (+) | MP80 (-) | MP160 (-) | (37~72) | MP (+) | 13 |
| 11 | MP-RNA (+) | MP80 (-) | MP160 (-) | >72 | MP (+) | 7 |
| 12 | MP-RNA (+) | MP80 (+) | MP160 (-) | (1~12) | MP (+) | 3 |
| 13 | MP-RNA (+) | MP80 (+) | MP160 (-) | (13~36) | MP (+) | 12 |
| 14 | MP-RNA (+) | MP80 (+) | MP160 (-) | (37~72) | MP (+) | 5 |
| 15 | MP-RNA (+) | MP80 (+) | MP160 (-) | >72 | MP (+) | 10 |
| 16 | MP-RNA (+) | MP80 (+) | MP160 (+) | (1~12) | MP (+) | 11 |
| 17 | MP-RNA (+) | MP80 (+) | MP160 (+) | (13~36) | MP (+) | 22 |
| 18 | MP-RNA (+) | MP80 (+) | MP160 (+) | (37~72) | MP (+) | 35 |
| 19 | MP-RNA (+) | MP80 (+) | MP160 (+) | >72 | MP (+) | 31 |
| 20 | MP-RNA (-) | MP80 (-) | MP160 (-) | (1~12) | MP (-) | 125 |
| 21 | MP-RNA (-) | MP80 (-) | MP160 (-) | (13~36) | MP (-) | 129 |
| 22 | MP-RNA (-) | MP80 (-) | MP160 (-) | (37~72) | MP (-) | 55 |
| 23 | MP-RNA (-) | MP80 (-) | MP160 (-) | >72 | MP (-) | 27 |
| 24 | MP-RNA (-) | MP80 (+) | MP160 (-) | (1~12) | MP (-) | 5 |
| 25 | MP-RNA (-) | MP80 (+) | MP160 (-) | (13~36) | MP (-) | 12 |
| 26 | MP-RNA (-) | MP80 (+) | MP160 (-) | (37~72) | MP (-) | 11 |
| 27 | MP-RNA (-) | MP80 (+) | MP160 (-) | >72 | MP (-) | 7 |
| 28 | MP-RNA (+) | MP80 (-) | MP160 (-) | (1~12) | MP (-) | 1 |
| 29 | MP-RNA (+) | MP80 (-) | MP160 (-) | (13~36) | MP (-) | 2 |
| 30 | MP-RNA (+) | MP80 (-) | MP160 (-) | >72 | MP (-) | 2 |
