# Supplementary figures and images for: Application value of antibody titres and RNA detection in the early prediction of Mycoplasma pneumoniae pneumonia in children: a retrospective study
Source: BMC Infect Dis. 2023 Apr 7;23:220. doi: 10.1186/s12879-023-08161-8 (PMC10082536; doi:10.1186/s12879-023-08161-8)

**Figure S1.** Diagnostic decision trees for children with MP.

*
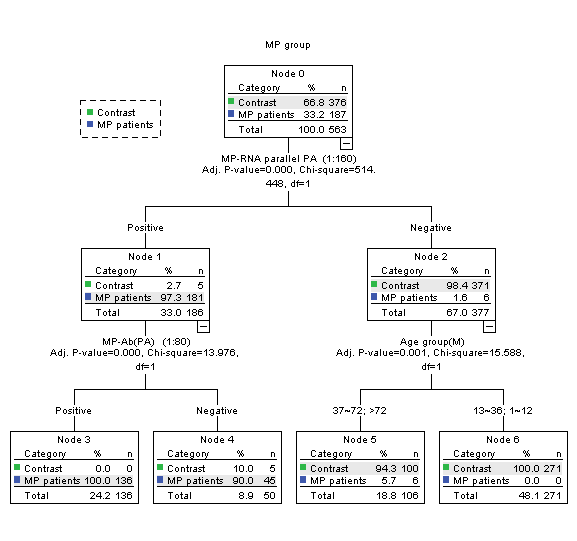
*

MP*, Mycoplasma pneumoniae*

Supplement: Supplementary file 3 — Figure S1. Diagnostic decision trees for children with MP. [file 12879_2023_8161_MOESM3_ESM.docx]
